# The emergence of two anti-phase oscillatory neural populations in a computational model of the Parkinsonian globus pallidus

**DOI:** 10.3389/fncom.2013.00173

**Published:** 2013-12-02

**Authors:** Robert Merrison-Hort, Roman Borisyuk

**Affiliations:** ^1^Centre for Robotics and Neural Systems, School of Computing and Mathematics, The University of PlymouthPlymouth, UK; ^2^Neural Networks Laboratory, Institute of Mathematical Problems in Biology, Russian Academy of SciencesPushchino, Russia

**Keywords:** Parkinson's disease, globus pallidus, oscillation, synchronization, HCN, downregulation, deep-brain stimulation

## Abstract

Experiments in rodent models of Parkinson's disease have demonstrated a prominent increase of oscillatory firing patterns in neurons within the Parkinsonian globus pallidus (GP) which may underlie some of the motor symptoms of the disease. There are two main pathways from the cortex to GP: via the striatum and via the subthalamic nucleus (STN), but it is not known how these inputs sculpt the pathological pallidal firing patterns. To study this we developed a novel neural network model of conductance-based spiking pallidal neurons with cortex-modulated input from STN neurons. Our results support the hypothesis that entrainment occurs primarily via the subthalamic pathway. We find that as a result of the interplay between excitatory input from the STN and mutual inhibitory coupling between GP neurons, a homogeneous population of GP neurons demonstrates a self-organizing dynamical behavior where two groups of neurons emerge: one spiking in-phase with the cortical rhythm and the other in anti-phase. This finding mirrors what is seen in recordings from the GP of rodents that have had Parkinsonism induced via brain lesions. Our model also includes downregulation of Hyperpolarization-activated Cyclic Nucleotide-gated (HCN) channels in response to burst firing of GP neurons, since this has been suggested as a possible mechanism for the emergence of Parkinsonian activity. We found that the downregulation of HCN channels provides even better correspondence with experimental data but that it is not essential in order for the two groups of oscillatory neurons to appear. We discuss how the influence of inhibitory striatal input will strengthen our results.

## 1. Introduction

Parkinson's disease is a neurodegenerative disorder which (amongst other symptoms) causes a range of movement-related disturbances such as tremor and slowness (bradykinesia). The primary pathology of the disease is the death of dopaminergic neurons in the basal ganglia (BG), specifically those in the substantia nigra pars compacta (SNc). Since the dopaminergic neurons in the SNc provide widespread innervation to the other regions of the basal ganglia, it is not surprising that their loss results in profound changes to neuronal activity in these regions. What is not yet understood is the precise mechanism by which abnormal neuronal activity arises as a result of dopamine loss—and how this activity relates to motor symptoms. One very successful hypothesis for this was the so-called “rate” hypothesis (DeLong, 1990), which held that motor areas of the basal ganglia are divided into two feed-forward pathways that transfer information from the cortex to the thalamus: a pro-kinetic “direct” and an anti-kinetic “indirect” pathway. According to this model, loss of dopamine input to the striatum upsets the balance of these two pathways, resulting in movement abnormalities. While the rate hypothesis makes predictions that have resulted in successful treatments, such as lesioning of hyperactive nuclei on the indirect pathway (Lozano et al., 1995; Gill and Heywood, 1997), more recent electrophysiological studies have demonstrated that the changes in neuronal activity that underlie Parkinsonian motor impairment are likely to be considerably more complex than those implied by the rate model [see Rubin et al. (2012) for review].

One aspect of pathological activity in the Parkinsonian basal ganglia that is under active investigation is the increase in synchronous oscillatory firing. Local field potential (LFP) recordings from the subthalamic nucleus (STN) of patients with Parkinson's disease show a clear increase in power in the β frequency band (10–30 Hz) when patients are off medication [reviewed in Eusebio and Brown (2009)], and the size of the reduction in β power that occurs with dopamine-replacement medication is positively correlated with the concomitant improvement in severity of anti-kinetic symptoms (Kühn et al., 2006). There are a number of reasons why widespread pathological oscillations may cause motor deficits, for example they may impair the ability to relay information (Mallet et al., 2008b). It has also been proposed that, in health, sporadic β oscillations act as a global signal for maintenance of the current motor activity (Jenkinson and Brown, 2011).

What is the neural basis for the exaggerated oscillatory activity of the Parkinsonian basal ganglia? One possibility is that the reciprocally-connected neurons of the excitatory STN and inhibitory globus pallidus (GP; homologous to the external globus pallidus in primates) act as a neural oscillator. Several computational studies have suggested that this is a plausible mechanism (Gillies et al., 2002; Terman et al., 2002; Holgado et al., 2010). *In vitro* work in slices containing only STN and GP neurons have also shown that oscillatory firing is indeed possible (Plenz and Kital, 1999), though only at frequencies much lower than the β band.

Another possible explanation for exaggerated BG β oscillations in Parkinson's disease is that dopamine acts to modulate the effects of rhythmic cortical activity on cortical-basal ganglia pathways, such that in conditions of reduced dopamine this network becomes pathologically entrained to cortical rhythms. Evidence for this comes from studies that have used signal processing techniques to attempt determine whether β band coherence between the cortex and basal ganglia is directed from cortex to STN or vice versa. Such studies have shown that, in patients with Parkinson's disease (Litvak et al., 2011) or Parkinsonian rodents (Sharott et al., 2005), the oscillations arise in the cortex and drive STN activity. Computational studies that investigate the synchronization of basal ganglia neurons in Parkinson's disease often consider the neurons to be phase oscillators, which either synchronize themselves Popovych and Tass (2012) or become synchronized through common external inputs Wilson et al. (2011).

Experiments using rodent models of Parkinson's disease provide compelling evidence that under Parkinsonian conditions the activity of neurons in the GP are much more susceptible to entrainment by cortical rhythms than in the healthy case. Under conditions of urethane anaesthesia, neurons in the GP of healthy rodents show uncorrelated tonic firing. However, in animals where Parkinsonism has been induced, either through chronic lesioning of the SNc with 6-hydroxydopamine (OHDA) (Ni et al., 2000; Magill et al., 2001) or acute inactivation of SNc projection fibers (Galati et al., 2009), the spiking activity of the majority of GP neurons becomes significantly correlated with cortical “slow wave activity” (SWA); this is the major cortical rhythm in the anaesthetized state and has a frequency of approximately 1 Hz. These experiments also reveal that, in the chronic lesioned case at least, the neurons in GP are split into two major groups, distinguished by whether they preferentially fire during the active phase (ECoG peaks) or inactive phase (ECoG troughs) of SWA in dopamine-deprived conditions. These will be referred to as the TA and TI groups, respectively. The underlying basis for this division is unknown, but the same division is seen in respect to cortico-pallidal synchronization that occurs transiently at β frequencies in response to sensory stimulation in OHDA lesioned rodents (Mallet et al., 2008a), which suggests that the same mechanism may be responsible for pathological entrainment in both behavioral states/frequency bands. If this is the case, then understanding this mechanism may lead to improved treatments for Parkinson's disease. Unfortunately we do not currently know the route through which oscillatory cortical input entrains the GP, although it is likely to involve the two major sources of synaptic input to GP neurons: the inhibitory medium spiny projection neurons of the striatum and excitatory STN neurons. Both receive widespread cortical inputs and both show increased firing during the peaks of SWA under Parkinsonian conditions in rodents (Magill et al., 2001; Tseng et al., 2001). Given that the majority of GP neurons belong to the TI group it has been suggested that cortical oscillations are most effectively relayed via the inhibitory striatum (Walters et al., 2007), but this view is challenged by the fact that the entrainment of GP neurons to SWA appears to be critically dependent on a functioning STN (Ni et al., 2000; Galati et al., 2009).

In this paper we test the hypothesis that the inhibitory network of GP neurons allows two anti-phase groups of oscillatory neurons to appear in response to rhythmic excitatory STN input only. To do this we consider a small neural network model of interconnected conductance-based GP neurons. Although the parameters of the neurons in this population are homogeneous, our simulations reveal a mechanism by which the two oscillatory groups can appear. This collective behavior is the result of a self-organization process that depends on the GP neurons' inhibitory dynamics and rhythmic STN modulation. We study the neural network model under healthy and Parkinsonian conditions and demonstrate a good correspondence between simulation results and experimental recordings. Special attention has been paid to studying the possible role of downregulation of hyperpolarization-activated cyclic nucleotide-gated (HCN) channels in this network, based on the effect that these channels appear to have on GP neurons' responses to synaptic input (Chan et al., 2004; Boyes et al., 2007) and the suggestion that oscillatory activity may not appear immediately after dopamine lesion and might instead depend on slower adaptive processes (Degos et al., 2009).

The structure of this paper is as follows. Section 2 describes our model including the cellular properties of model GP neurons, how STN neuron activity was generated, the nature of synaptic connectivity and our proposed model for HCN downregulation. Section 3 describes the results of simulations and demonstrates that model GP neurons behave realistically both in isolation and as part of a network. It also shows how the network's activity changes under simulated Parkinsonian conditions in a way that is similar to the results of previous biological experiments. Section 4 examines results in the context of what has been seen in animal models of Parkinson's disease and discusses what the results might mean in terms of potential improvements to treatments for the disease.

## 2. Materials and methods

**Figure 1:**
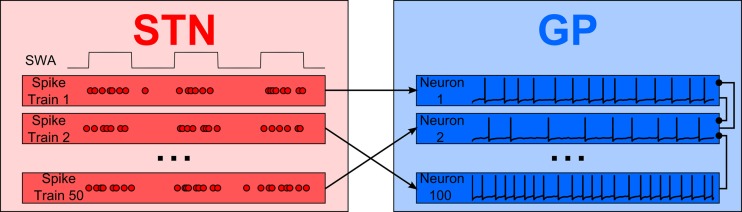
**An overview of the model**. Integrate-and-fire STN neurons, modulated by an approximately 1 Hz rhythm, provide excitatory synaptic input to a population of GP neurons. Inhibitory local synaptic connections between GP neurons have random connectivity.

Figure 1 shows a simple representation of the neural network model which includes a population of 100 interconnected GP neurons (right panel, blue) which receive excitatory synaptic input from 50 STN neurons (left panel, red). Each GP neuron is described by a detailed single compartment conductance based model of the Hodgkin–Huxley type with inhibitory connections from other GP neurons. The STN neurons are described by a simple enhanced leaky integrate-and-fire model. Neurons in the STN population are not connected to each other but they are modulated by a common cortical slow-wave rhythm and make excitatory synapses onto GP neurons.

### 2.1. Model GP neurons

The model GP neurons are of standard Hodgkin–Huxley type, with a single compartment per neuron. We included ten voltage-gated ionic channels as in the multicompartmental modeling work of Günay et al. (2008): fast and slow delayed rectifying K_+_ (Kv3 and Kv2, respectively), fast and slow A-type K_+_ (Kv4_fast_, Kv4_slow_), M-type K_+_ (KCNQ), fast spike-producing Na_+_ (NaF), persistent pacemaking Na_+_ (NaP), hyper-polarization activated Ca_2+_ (HVA), and fast and slow mixed-conductance hyperpolarization-activated channels (HCN_fast_, HCN_slow_). For simplicity our model does not include calcium-gated potassium “SK” channels, as these channels' most significant effect on the activity of GP neurons appears to be a lengthening of spike afterhyperpolarization (AHP) (Deister et al., 2009), and we were able achieve physiologically realistic AHPs without this channel. Equation 1 describes how the membrane potential (V) of a model GP neuron evolves in time.

(1) $$\begin{array}{l} C\frac{dV}{dt}=g_{leak}(E_{leak}-V)+I_{naf}+I_{nap}\\ \;+I_{kv2}+I_{kv3}+I_{kv4f}+I_{kv4s}+I_{kcnq}\\ \;+I_{hva}+I_{hcnf}+I_{hcns}+I_{syn}+I_{ext} \end{array}$$

Here *C* and *g*_*leak*_ are the total membrane capacitance and leak conductance and *E*_*leak*_ is the reversal potential of the leak channels. Values for these parameters are given in Table 1. *I*_*syn*_ is the total synaptic current received by the neuron (see below). *I*_*ext*_ is an externally applied current that was only non-zero when testing the response of individual GP neurons to current injections. The remaining currents correspond to the voltage-dependent channels, each of which has an activation gate (represented by the state variable *m*) and, for most channels, an inactivation gate (state variable *h*). Two channels have slow inactivation gates (*s*) in addition to their activation and inactivation gates. Equation 2 shows the current due to a channel with all three gates:

(2) $$I=m^{\mu }h^{\rho }s^{\phi }g_{X}(E_{X}-V)$$

**Table 1:** **Passive membrane parameters and channel conductances for the model GP neurons**.

**Parameter**	**Value**	**Units**
C	141.6	pF
g_leak_	4.012	nS
E_leak_	−60.0	nS
g_naf_	5900.0	nS
g_nap_	17.7	nS
g_kv2,3,4f,4s_	590.0	nS
g_kcnq_	1.77	nS
g_hva_	1.77	nS
g_hcaf,s_	177.0	nS
E_k_	−80.0	mV
E_na_	55.0	mV
E_ca_	120.0	mV
E_hcn_	−30.0	mV

Here *E*_*X*_ is the reversal potential of the channel, *g*_*X*_ is the maximum conductance of the channel, and μ, ρ and ϕ are integers that give the relative numbers of gating molecules. Table 2 shows which channels contain each gate type and the corresponding values of μ, ρ and ϕ. Note that the parameters governing the dynamics of each gate vary from channel to channel. Since we use the same equations and parameters for channel gates as Günay et al. (2008) we do not reproduce these here and instead refer to the supplementary material of that paper where they are listed.

**Table 2:** **The relative proportion of gating molecules of each type for each channel in the model GP neurons**.

**Channel**	μ	ρ	ϕ
NaF	3	1	1
NaP	3	1	1
Kv2,3,4_fast_, 4_slow_	4	1	–
KCNQ	4	–	–
HVA	1	–	–
HCN_fast_, HCN_slow_	1	–	–

We could not directly use the channel maximum conductance parameters from Günay et al. (2008), since this was a multicompartmental model and the conductances varied widely between the different compartments. Instead we adjusted the channel conductances so that our model neurons exhibited intrinsic pacemaking and displayed realistic responses to depolarizing and hyperpolarizing current injections. The chosen conductances are shown in Table 1. In order to generate a range of intrinsic pacemaking frequencies we applied Gaussian noise to the maximum conductances of the persistent sodium and HCN channels (*g*_*nap*_, *g*_*hcnf*_, and *g*_*hcns*_). The mean values are given in Table 1 and the standard deviation was 50% of the mean value for NaP and 30% for both HCN channels.

It has been proposed (Chan et al., 2011) that a homeostatic mechanism may reduce the intrinsic firing rate of GP neurons via downregulation of HCN channels in response to burst firing. Our model includes a possible mechanism for this downregulation by allowing the maximum conductance of HCN channels to decrease. This occurs during periods of elevated firing rate, which are indicated by high intracellular calcium concentration ([*Ca*]). In order to model the dynamics of this calcium concentration we use the equations from Terman et al. (2002). The rate of change of intracellular calcium is given by Equation 3:

(3) $$\frac{d[Ca]}{dt}=\epsilon (I_{hva}-k_{Ca}[Ca])$$

Here ∈ represents calcium buffering and has the value 10^−4^ $\frac{Ms}{CL}$, while *k*_*Ca*_ is the calcium pump rate and has the value 15.0 $\frac{CL}{Ms}$ [parameter values from Rubin and Terman (2004)]. *I*_*hva*_ represents the instantaneous current due to HVA channels (these are the only Ca_+_ channels in the model). The maximum conductance of HCN channels remains constant during time steps and is adjusted between steps when [*Ca*] > T_*HCN*_, where *T*_*HCN*_ is the threshold above which downregulation occurs. The amount by which the conductance is adjusted takes the form of a sigmoid curve and is given in Equation 4.

(4) $$g_{HCN}(t+\Delta t)=max\left[0,g_{HCN}(t)-\frac{k_{HCN}\Delta t}{1+exp(\frac{\theta -[Ca](t+\Delta t)}{\sigma })}\right]$$

Here *k*_*HCN*_ is the maximum conductance change that can occur in one step, θ is the level of intracellular calcium that gives half the maximum change, and σ is the slope of the sigmoid. We do not have any data from biological experiments to suggest values for the downregulation parameters. Since we hypothesize that downregulation mostly only occurs during fast burst firing under Parkinsonian conditions, we chose parameters such that, in healthy conditions, downregulation only occurred in the very fastest firing GP neurons. The values of *k*_*HCN*_ and σ that we chose give a fairly rapid reduction in maximum HCN conductance in response to elevated firing rates. The downregulation parameters that we chose are: *T*_*HCN*_ = 0.2, *k*_*HCN*_ = 6 × 10^−4^, σ = 0.1, θ = 0.5.

Note that our model GP neurons are supposed to represent those in the rodent GP. This nucleus is usually considered to be equivalent to the so-called “external” pallidus (GPe) in primates.

### 2.2. Model STN neurons

Since the aim of this study is to investigate the effects of rhythmic STN input upon the neurons of the GP, we did not model STN neurons to the same level of biological detail as GP neurons. Instead, to simulate the SWA-modulated bursting of STN neurons that occurs under urethane anaesthesia we use an enhanced integrate-and-fire generator of neural activity, as described in Borisyuk (2002). The STN neurons include an exponentially decaying threshold, accumulation of membrane potential, stochastic noise and an absolute refractory period. Aside from approximating the SWA modulation of STN activity our model does not include any synaptic inputs to the STN. In particular we do not include the GP-STN projection because experiments in urethane-anaesthetized rats have shown that the changes in firing rate and pattern that occur in the STN following OHDA-lesion are not dependent on synaptic input from the GP (Hassani et al., 1996).

**Figure 2:**
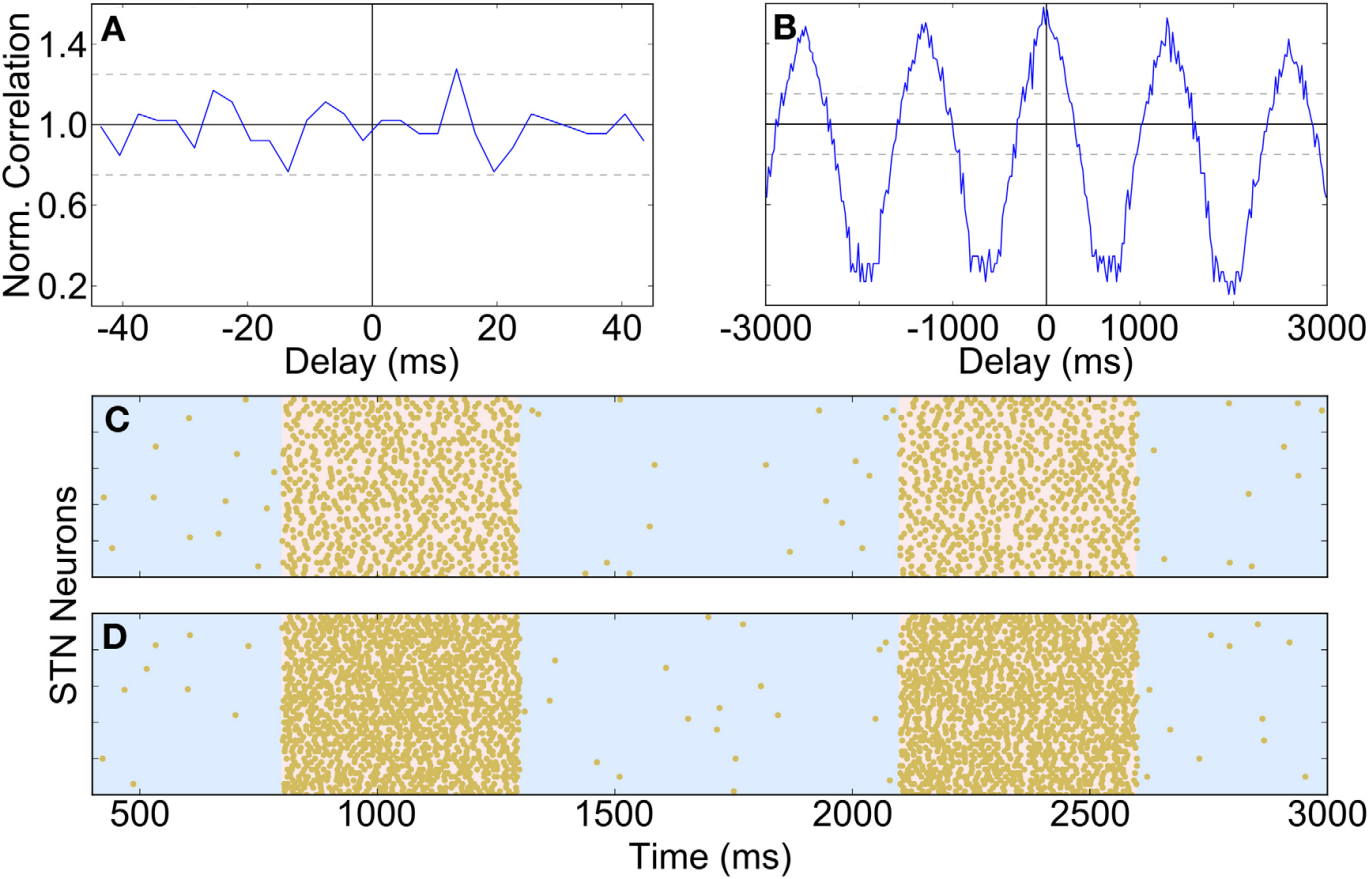
**Properties and spiking activity of STN neurons. (A)** Example cross-correlation between two STN neuron spike trains on a short time window (30 bins, 3 ms each) shows independent firing. **(B)** Longer time window reveals slow (1300 ms) oscillations (400 bins, 20 ms each). **(C)** Spiking activity of STN neurons under healthy conditions. **(D)** Increased intensity of active-phase firing under Parkinsonian conditions. In **(C,D)** the background is shaded to show the active (pink) and inactive (blue) phases of the SWA cycles. **(A,B)** are normalized using the procedure described in Brillinger (1976): if *X*_*i*_ is the unscaled spike count in histogram bin *i* then the scaled value *X*'_*i*_ is given by: *X*'_*i*_ = $\frac{TX_{i}}{2hN_{A}N_{B}}$, where *T* is the total simulation time, *h* is half the width of a cross-correlation bin and *N*_*A*_ and *N*_*B*_ are the total spike counts for each spike train. A normalized value of 1 indicates that there is no correlation at a particular delay, while deviations from 1 indicate positive or negative correlations. The horizontal bars show the 95% confidence interval for significant correlations (Brillinger, 1976).

During urethane anaesthesia STN neurons display uncorrelated bursting activity that is modulated by a slow rhythm (Magill et al., 2001) which, for the purposes of this paper, we assume arises from excitatory cortical inputs. Since firing is uncorrelated, each STN spike train is generated independently using a procedure that results in spiking activity that is similar to the spiking activity of real neurons. The generated spike trains contain activity that is oscillatory with a period of 1300 ms (≈0.8 Hz), where cycle is made up of an 800 ms “inactive” phase and a 500 ms “active” phase. The spike trains are constructed by alternately sampling from two intermediate spike trains, one with slow irregular firing and one with fast irregular firing (for the inactive and active periods, respectively). The average firing rates are 0.5 Hz for the inactive period and 30 Hz for the active period; under simulated Parkinsonian conditions the firing frequency during the active phase is increased to 60 Hz. Figure 2 shows cross-correlations between two STN spike trains which demonstrate that within the active bursting period activity is not correlated (A), but that there is strong common modulation at 0.8 Hz (B). Figures 2C,D are raster plots of the generated spiking activity of 50 STN neurons in healthy and Parkinsonian conditions, respectively, demonstrating a clear increase in firing frequency during the active phase in the Parkinsonian case.

### 2.3. Synaptic connectivity

Each model neuron contains two variables, *o*(*t*) and *c*(*t*), which represent synaptic opening and closing, respectively. When a neuron spikes (defined by the membrane potential crossing 0 mV in the positive direction), both variables are step-increased by 1. Following this, the variables decay exponentially to zero according to different time constants τ_*o*_ and τ_*c*_, respectively (Eq. 5). Since τ_*o*_ < τ_*c*_, a transient synaptic current arises in all post-synaptic neurons following a spike, according to Eq. 6:

(5) $$\frac{do}{dt}=-\frac{1}{\tau_{o}}o\;\frac{dc}{dt}=-\frac{1}{\tau_{c}}c$$

(6) $$I_{syn}=(c-o)g_{Syn}(e_{rev}-V)$$

Here *e*_*rev*_ is the reversal potential of the synapse (mV) and *V* is the membrane potential of the post-synaptic neuron (mV). The value of parameters *e*_*rev*_, τ_*c*_ and τ_*o*_ vary based on the type of the neuron (glutamatergic STN or GABAergic GP). *g*_*Syn*_ denotes the maximum unitary conductance of a synapse (nS) and its value for a particular synapse is drawn from a Gaussian distribution. The mean of this distribution was *g*_*SG*_ for STN-GP synapses and *g*_*GG*_ for intra-GP synapses and the standard deviation was 30% of the mean in both cases.

For the intra-GP inhibitory synapses we used synaptic time constants xτ_*o*_ = 5 ms and τ_*c*_ = 40 ms from unpublished current-clamp recordings of GP-GP IPSPs taken from slices containing rat GP, cortex and striatum [Alon Korngreen, personal communication]. Similarly, we chose *g*_*GG*_ = 0.5 nS which gives a peak IPSP of 0.5 mV (measured as deflection away from a holding potential of −65 mV during injection of hyperpolarizing current) to match the same experimental recordings. We used a standard GABA reversal potential of −80 mV. Connectivity between GP neurons was uniformly random, with each neuron inhibiting 20 others (no self-connections).

Anatomical data regarding the structure of the STN-GP projection is currently lacking. However, it is clear that there are many fewer STN neurons than GP neurons and that each GP neuron only samples the activity of a small proportion of STN neurons (Jaeger and Kita, 2011). We therefore arbitrarily chose to model 50 STN neurons, each of which makes excitatory synapses onto two randomly selected GP neurons. The time constants of STN-GP synapses in the model are τ_*o*_ = 0.2 ms and τ_*c*_ = 60 ms, based on the recordings shown in Loucif et al. (2005). The average maximum synaptic conductance (*g*_*SG*_) used for the healthy case was chosen to be just low enough such that the majority of GP neurons didn't show significant entrainment to the SWA rhythm and we investigated the effects of increasing the value in the Parkinsonian case.

### 2.4. Modeling of Parkinsonism

We simulate the OHDA-lesioned (Parkinsonian) rat basal ganglia by making three changes to the model's parameters: (i) faster STN firing during the active phase of SWA (Figure 2D) (ii) increased STN-GP synapse strength and (iii) increased intra-GP inhibition strength. Although the changes that occur to functional connectivity in the basal ganglia in Parkinson's disease are currently under active investigation, there is experimental support for facilitation of both GP-GP (Johnson and Napier, 1997) and STN-GP (Johnson and Napier, 1997; Hernández et al., 2006) synapses. Similarly, under urethane anaesthesia it has been shown that spiking in the STN continues to be modulated by the cortical SWA rhythm, but that its firing becomes more intense during the active period (Magill et al., 2001; Galati et al., 2009).

### 2.5. Catergorization of neuron activity

We used a simple method to catergorize GP neurons as being of type TA (in-phase with active SWA), TI (in-phase with inactive SWA), NM (not modulated by SWA) or QU (quiet). Each spike fired by the neuron to be catergorized is represented by a complex number that indicates its phase in relation to SWA. The sum of these complex numbers then gives an indication of the average phase, ω, as shown in Eq. 7.

(7) $$\omega_{k}=\sum_{s\in S_{k}}e^{i\theta (s)}$$

Here *S*_*k*_ is the set of spike times for neuron *k* (0 ≤ *k* < 100) and θ(*s*) is the phase of SWA at time *s* (0 ≤ θ(*s*) < 2 π). The argument of the complex number ω_*k*_ indicates the average SWA phase at which neuron *k* fires, while its modulus provides an indication of how strongly SWA-modulated the firing is. Normalizing the modulus by the number of spikes gives a confidence measure *c*_*k*_ = $\frac{\left|\omega_{k}\right|}{\left|S_{k}\right|}$, where *c*_*k*_ = 0 indicates that spikes did not fire preferentially at any one phase and *c*_*k*_ = 1 indicates that every spike occurred at exactly the same phase. After visual inspection of spike trains, we decided to catergorize neurons with confidence *c* < 0.1 as NM. We catergorized neurons with *c* ≥ 0.1 as either TA or TI based on whether the average phase was during the active or inactive part of the SWA cycle. Neurons that fired fewer than one spike every SWA cycle on average were catergorized as QU.

### 2.6. Implementation details

We simulated the model using custom written software developed by R Merrison-Hort. This software is written in C and uses the adaptive Runge-Kutta-Felberg ODE solver routine from the GNU Scientific Library (version 1.15). Absolute and relative error tolerances of 10^−5^ and a maximum step size of 1 ms were used for all simulations. To analyse the results we used scripts written in the Python (2.7) programming language with routines from the NumPy (1.6.2) and SciPy (0.11.0rc1) libraries. For each set of parameters twelve simulations were performed, with different random neural connectivity, STN spike trains and parameter noise (as described above) in each simulation.

All reported mean values are given with their standard deviation.

## 3. Results

### 3.1. Model GP neurons behave realistically under healthy conditions

**Figure 3:**
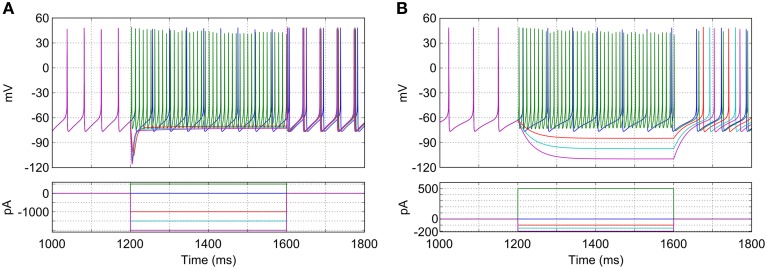
**Response of a typical isolated model GP neuron to different current injections. (A)** Neuron with normal HCN channel density. Depolarizing current causes fast, regular spiking (green trace), while hyperpolarizing current reveals a sag in membrane potential and rebound firing (red, cyan, and purple traces). With no current injection the neuron fires regularly at approximately 22 Hz (blue trace). **(B)** Neuron with HCN channels removed. Sag in membrane potential is lost and pacemaking is slowed. Note the difference in scale for the injection currents between **(A)** and **(B)**.

The characteristics of the model GP neurons qualitatively match that those of real rodent GP neurons in a number of key ways and are illustrated in Figure 3A. When no synaptic or injected currents are present (dark blue trace in Figure 3A), most model neurons (96%; 481/500) pacemake at a range of frequencies (23.6 Hz ± 4.0). Depolarizing current injections increase the frequency of firing (green trace), with very high frequencies possible (up to approximately 200 Hz). Hyperpolarizing current injections result in a prominent and transient “sag” in membrane potential (red, cyan and pink traces). The first spike after hyperpolarizing current is removed occurs after a similar delay regardless of the size of the injected current. These properties match those seen in experiments with slices of rodent GP (Chan et al., 2004; Bugaysen et al., 2010).

The mixed-conductance HCN channels play an important role in the activity of the model GP neurons and their response to hyperpolarizing input. The combination of a reasonably depolarized reversal potential (−30 mV) (Lüthi and McCormick, 1998) and activation at hyperpolarized membrane potentials (lower than −60 mV) means that these channels act to return neurons to spiking threshold faster after hyperpolarizing current (or inhibitory synaptic input) is removed. Figure 3B shows how the simulated blockade of HCN channels affects the activity of a model GP neuron. When HCN channels are removed (*g*_*hcnf,s*_ = 0), the average pacemaking frequency decreases to 15.8 Hz ± 2.5 and 12% (58/500) of neurons do not pacemake. Without HCN channels the membrane potential sag is no longer seen, and hyperpolarizing current has a much stronger effect on membrane potential. The time between the removal of hyperpolarizing current and the return of spiking is also much longer, and much more sensitive to the hyperpolarization level. These results agree with previous work that has investigated the role of HCN channels using mouse GP slices and multicompartmental simulations (Chan et al., 2004).

### 3.2. Healthy network activity

**Figure 4:**
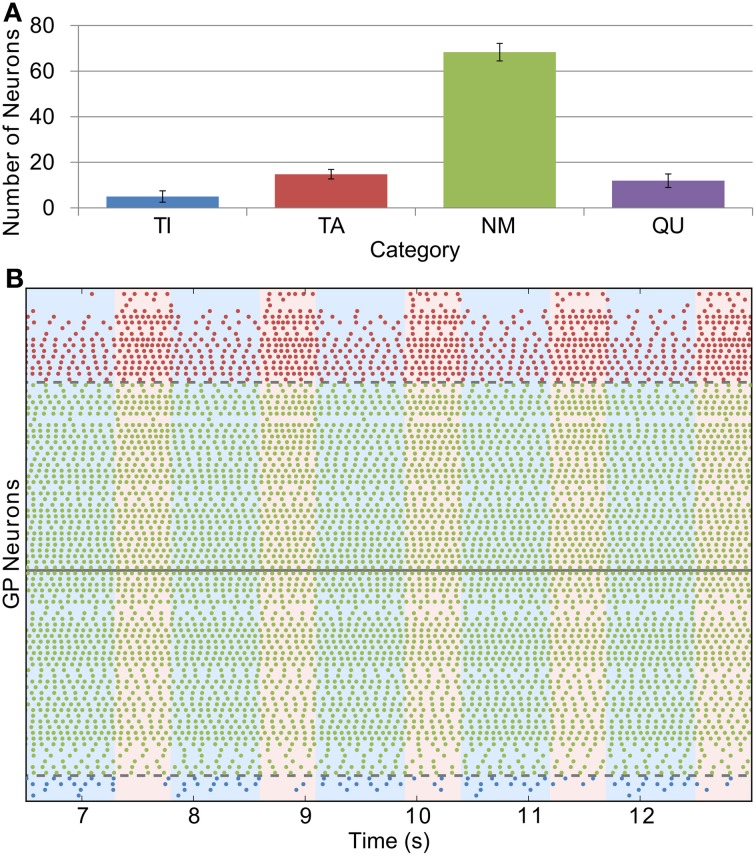
**Catergorization and activity of neurons under healthy conditions. (A)** The average number of GP neurons in each category across all trials (*n* = 12), showing that most cells do not display prominent modulation by SWA (error bars show standard deviation). **(B)** Raster plot showing the spiking activity of TI, TA and NM neurons in one representative trial. The spike trains above the solid gray line are from neurons whose average spike phase is in the active part of the SWA cycle (light pink shaded background), whilst those below the line have average phases in the inactive part of SWA (light blue shaded background). The spike trains are ordered such that those closer to the solid gray line have lower confidence measures than those further away. The dashed gray lines show the confidence measure boundaries that divide neurons classified as NM from those classified as TI or TA (this boundary is set at 0.1).

Whilst we were able to base STN firing rate and the conductance of GP-GP synapses directly on experimental evidence, we could only do this indirectly with the STN-GP synaptic conductance (*g*_*SG*_). We chose a value of 0.1 nS for this parameter in the healthy case as this gives similar proportions of neurons in the TI, TA and NM groups as seen in experiments. Figure 4A shows this distribution [cf. Figure 2A in Mallet et al. (2008b)] and Figure 4B shows the spiking activity of the TI, TA and NM neurons in one trial. A small proportion of neurons (9.9 % ± 2.1) are catergorized as QU because they fire spikes rarely or not at all; we are not sure if this is a biologically accurate result as such neurons may have been excluded from the results of electrophysiological studies. The majority of neurons (68.3% ± 3.9) are catergorized as NM and neurons in this group spike with an average firing rate of 12.3 Hz ± 3.3 and coefficient of variation (CV) of 0.12 ± 0.04. These statistics are in good agreement with those of neurons recorded from mice GP slices by Chan et al. (2004) (firing rate 12.5, CV 0.18). However, in contrast to these experimental results, which found no effect on firing rate or CV after blocking GABA_A_ receptors, we would expect the average firing rate of the neurons in our model to increase slightly with inhibition blocked, as the average firing rate in the network is lower than the average pacemaking frequency of isolated model neurons. The average firing frequencies in the (small) TI and TA groups were 3.7 Hz ± 1.8 and 10.7 Hz ± 4.7, respectively.

### 3.3. Parkinsonian network activity

**Figure 5:**
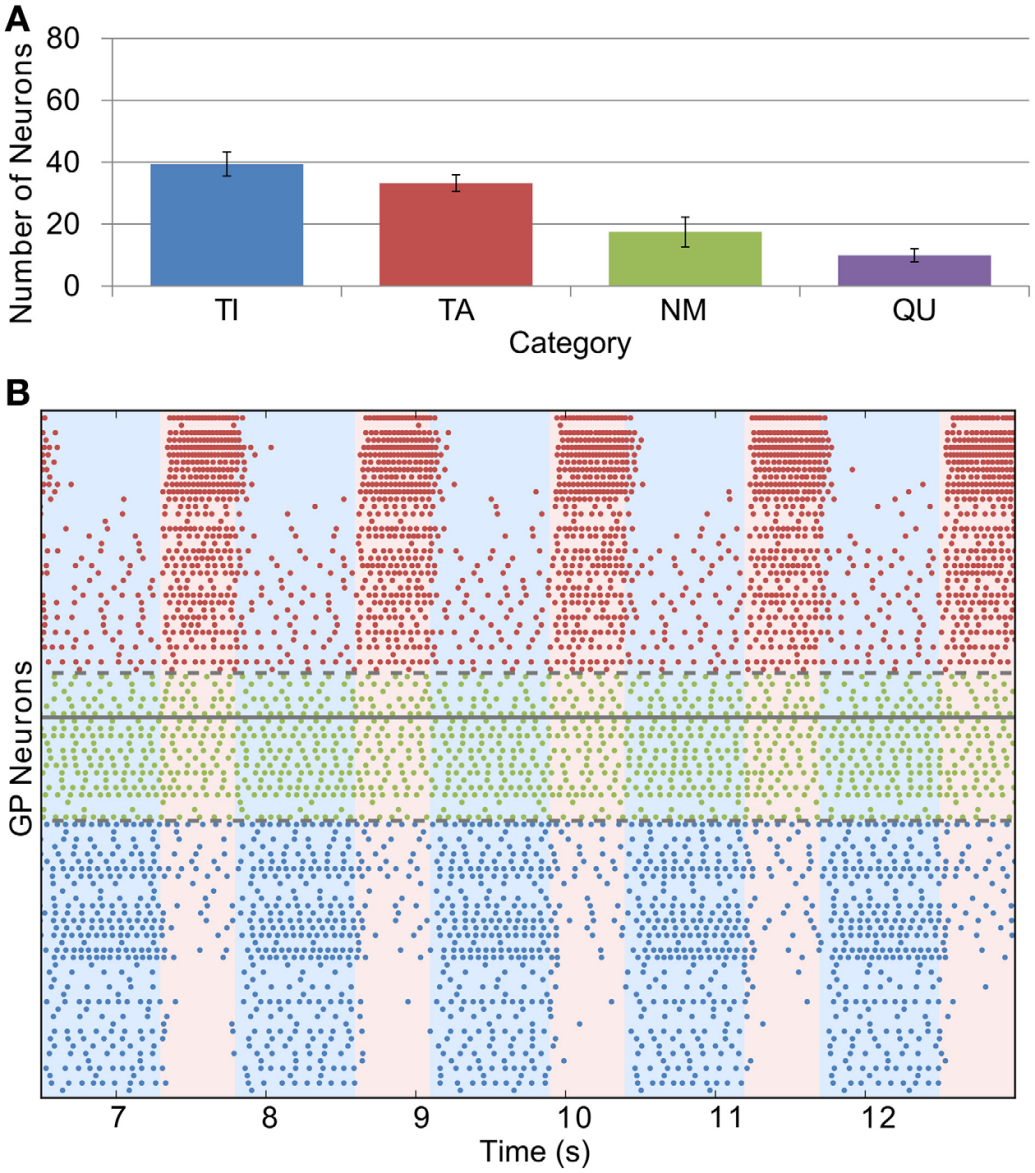
**Catergorization and activity of neurons under Parkinsonian conditions. (A)** The average number of GP neurons in each category across all trials (*n* = 12), showing that most neurons start to display SWA-modulated firing patterns, either in-phase (TA) or anti-phase (TI). **(B)** Raster plot of Parkinsonian GP neuron activity (description as in Figure 4B).

The effects of dopamine lesion were simulated in the model by an increased intensity of STN firing and increased strength of STN-GP excitation and intra-GP inhibition. These changes have a profound effect on activity in the model GP that is similar to what is seen in experiments. As Figure 5 shows, most neurons begin to preferentially fire during either the active or inactive phase of the SWA. In order to see proportions of TA and TI neurons that were similar to *in vivo* results it was necessary to double the strength of STN-GP and GP-GP synapses (*g*_*SG*_ = 0.2 nS, *g*_*GG*_ = 1.0 ns).

**Figure 6:**
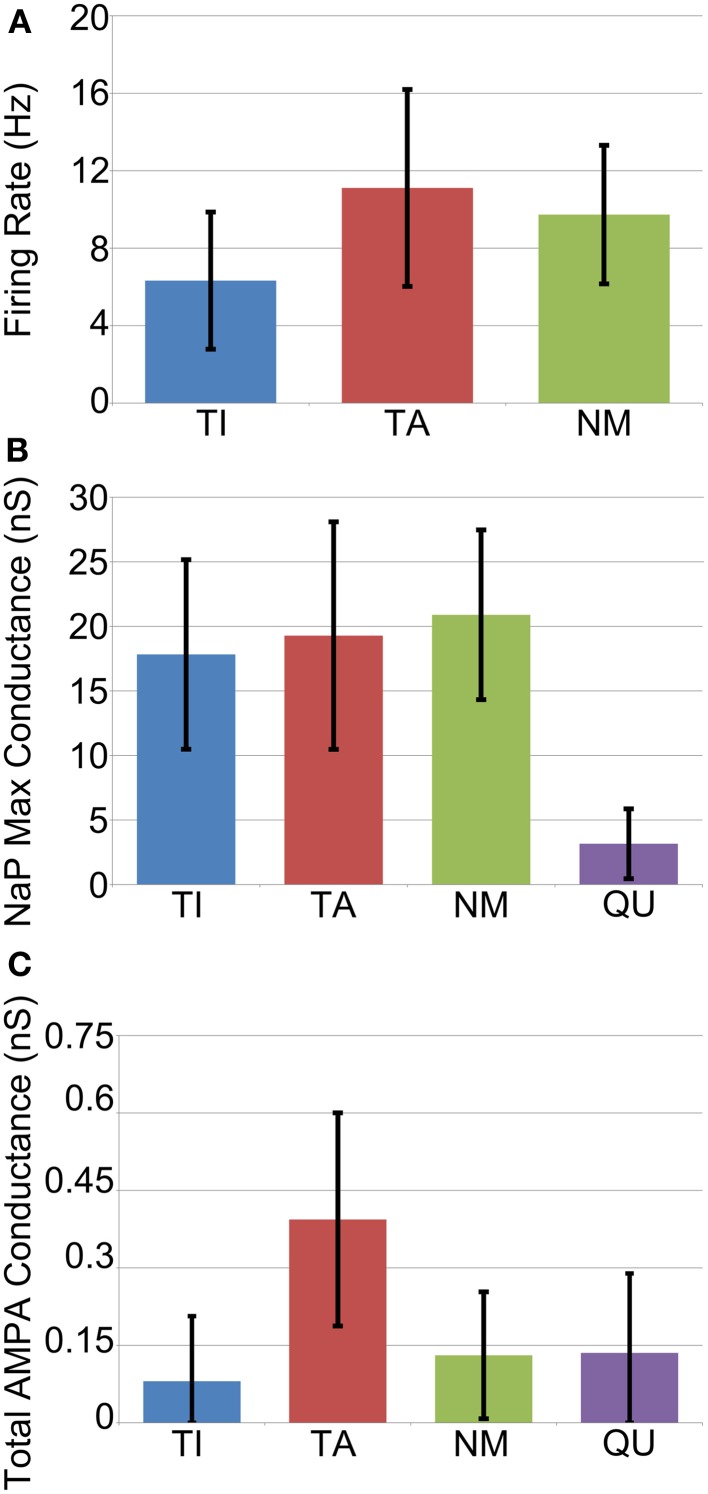
**Average cell properties by categorization across all GP neurons from all trials (*n* = 1200). (A)** Average firing rate is rather variable but is in general slightly lower for TI neurons. **(B)** Maximum conductance of the persistent sodium channel (NaP) which underlies pacemaking. Quiet neurons can be easily categorized as those with very low NaP conductance. **(C)** Average total maximum conductance due to excitatory synapses. TA neurons receive more excitation on average, but it is highly variable.

The average firing frequency of NM neurons decreased slightly under Parkinsonian conditions while the average firing rates of the TI and TA groups increased to 6.3 ± 3.6 and 11.1 Hz ± 5.1, respectively (Figure 6A). Although the variance of these statistics is fairly large, there does appear to be a trend for different firing rates between the different groups that is not seen *in vivo* (Magill et al., 2001). This difference is perhaps not too surprising given our simplistic and somewhat arbitrary choices for STN-GP and GP-GP connectivity.

In order to investigate the factors that determine whether a neuron becomes TA, TI, NM or QU we examined the following statistics of each neuron: maximum conductance of the NaP persistent sodium channel; initial (before downregulation) maximum conductance of fast and slow HCN channels; total maximum conductance of all excitatory (AMPA) synapses from STN neurons; total maximum conductance of all inhibitory synapses from other GP neurons. In general these statistics were remarkably similar between each of the groups, with two exceptions. Firstly, quiet (QU) neurons have, on average, much lower maximum conductances for their NaP channel (Figure 6B). These channels underlie autonomous pacemaking (Mercer et al., 2007) and the intrinsic pacemaking frequency is strongly dependent on the value of the NaP maximum conductance. Since QU neurons have low NaP conductance they are likely to pacemake very slowly or not at all, and it appears (from examination of voltage traces) that the incoming inhibition from other GP neurons is sufficient to prevent them from ever firing. Secondly, TA neurons receive on average more excitatory synaptic input from the STN than the other groups (Figure 6C). This result was expected, since STN firing occurs during the active phase of SWA and so it is not surprising that those GP neurons that receive more STN input also fire preferentially during the active phase. In general, however, the simple statistics we examined about membrane properties and synaptic connectivity are not enough to determine which group a particular neuron will fall into. Predicting the classification of a neuron involves knowing the classification of the other GP neurons that it receives inhibition from. This makes the problem complex and extremely difficult to resolve a priori. Instead, when the network is simulated, a dynamic process takes place in which the network self-organizes its activity into the different groups of neurons.

HCN channel downregulation plays a significant, but not essential, role in the emergence of the different groups of neurons in our model. Without this mechanism, many neurons in the TA group continue to fire during the inactive phase due to their intrinsic pacemaker properties. Although this inactive-phase firing is slower than their active-phase firing (due to reduced excitatory input), it is still a source of inhibitory input to other GP neurons and may silence or slow the firing of some which may otherwise be catergorized as TI. With the HCN downregulation mechanism those neurons that receive the most excitatory STN input, and therefore fire at the fastest rate during the active period, will have their maximum HCN conductances reduced. This reduction has the effect of decreasing the intrinsic pacemaking frequency and increasing the hyperpolarization that occurs in response to inhibitory input. To quantify the effect on pacemaking frequency, we ran four simulations using the Parkinsonian parameter values for a period of time long enough for downregulation to take effect (6.5 s) and then removed all synapses and recorded firing rates. The average pacemaker frequency after downregulation was 16.9 Hz ± 2.1, a clear reduction from the normal pacemaker frequency of our model neurons (23.6 Hz ± 4.0). The proportion of QU neurons after downregulation was 2%, lower than the 4% that we would expect based on the normal pacemaker properties, but we attribute this to statistical noise due to the rather small sample sizes.

**Figure 7:**
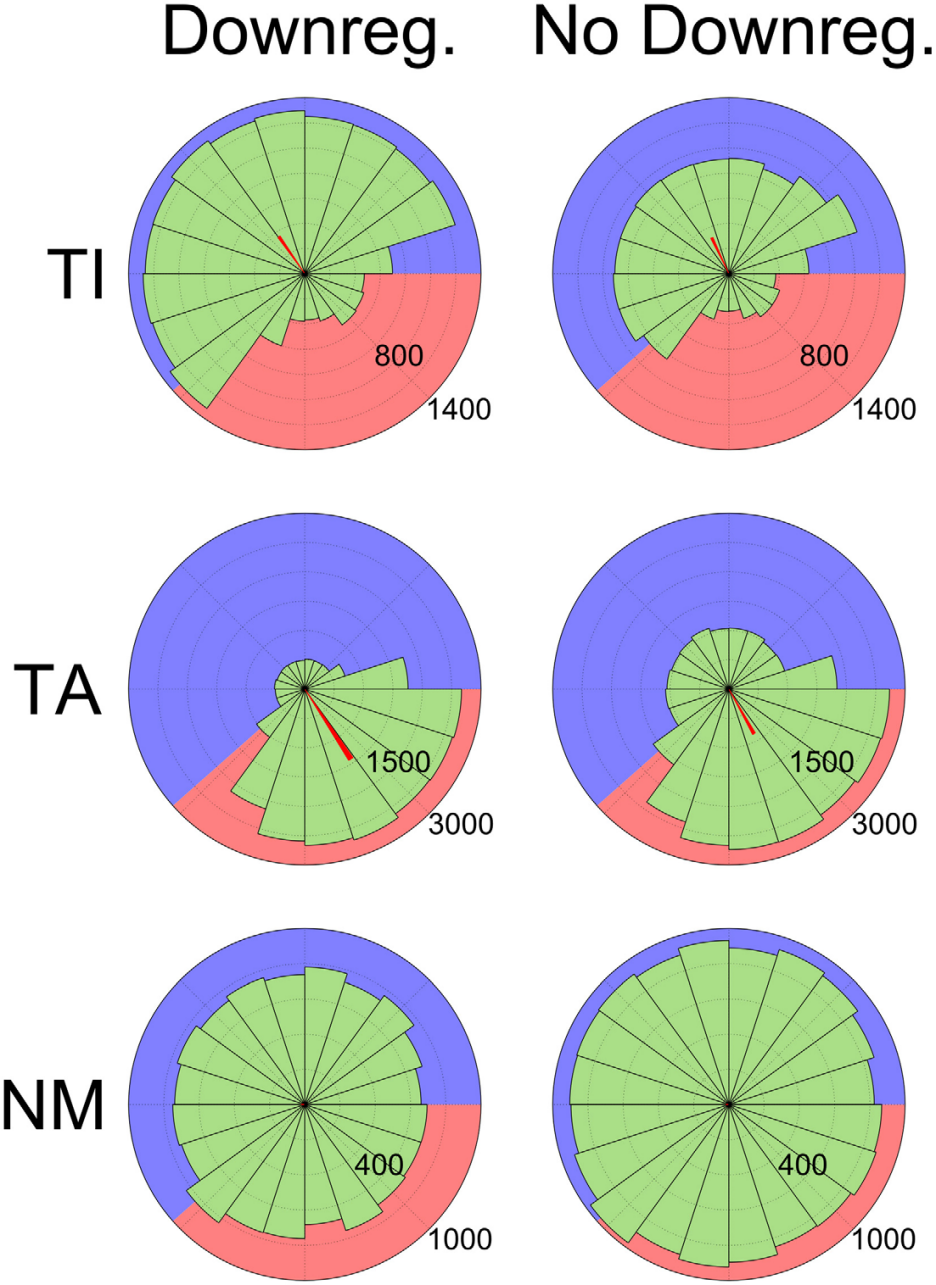
**Phase histograms showing the distribution of spikes relative to SWA phase grouped by categorization (**Top:** TI, **Middle:** TA, **Bottom:** NM), demonstrating the effects of HCN downregulation**. Numbers indicate the number of spikes in each bin (65 ms bin width). Data are shown from two sets of 12 simulations, one with the HCN downregulation mechanism enabled (left) and one with it disabled (right). The background of each diagram is shaded to illustrate the active (pink, 500 ms) and inactive (blue, 800 ms) parts of the SWA cycle. Downregulation reduces TA neuron firing during the inactive phase **(Middle)**, increases TI firing during the inactive phase **(Top)** and decreases NM firing **(Bottom)**. Spikes from the latter half of simulations (6.5 s out of 13 s) from all neurons (except those categorized as QU) were used to generate the diagrams. The orientation of the red bars shows the average spike phase and their length shows the phase confidence measure (ω) as a proportion of the total radius.

These changes to pacemaking affect the competition dynamics during the inactive phase and mean that most TA neurons are no longer able to fire at all during this phase. Although the proportion of neurons classified as either TA or TI is similar with or without HCN downregulation (72.7% ± 5.4 normally, 68.7% ± 5.0 without downregulation), TA neuron firing is much more clearly restricted to the active phase with downregulation. This is seen in the average confidence measure (ω) of TA neurons, which is 0.44 ± 0.22 with HCN downregulation and 0.32 ± 0.18 without. The effect is also shown by phase diagrams showing the spiking activity of typical neurons (Figure 7). In these plots the background is shaded to show the active (pink) and inactive (blue) parts of the SWA cycle, showing that TI neurons fire preferentially in the inactive part and TA neurons fire preferentially in the active part. The red bars show the average spike phase and their length indicates the confidence measure as a proportion of the total radius.

### 3.4. Larger networks

The results described above were from simulations with 100 GP neurons, each of which made 20 inhibitory synapses onto 20 other (randomly chosen) GP neurons. This level of coupling (≈ 20%) is probably much higher than what is seen in the real GP (Sadek et al., 2007). We ran several simulations, using Parkinsonian parameter settings, where the coupling proportion was decreased by scaling up the number of GP and STN neurons but keeping the number of synapses that each neuron made constant. In the first set of simulations we used 200 GP neurons and 100 STN neurons and in the second set we used 300 GP neurons and 150 STN. These give GP-GP coupling levels of 20/199 ≈ 10% and 20/299 ≈ 7%, respectively. In each case we ran three simulations. For the simulations with 200 GP neurons there were an average of 71±2.8 TI neurons and 73.7±1.7 TA neurons. For the simulations (*n* = 3) with 300 GP neurons there were an average of 122±4.1 TI neurons and 101.3±2.5 TA neurons. These proportions (particularly in the latter case) are similar to the proportions in the smaller network (see Figure 5).

### 3.5. Other frequency bands

**Figure 8:**
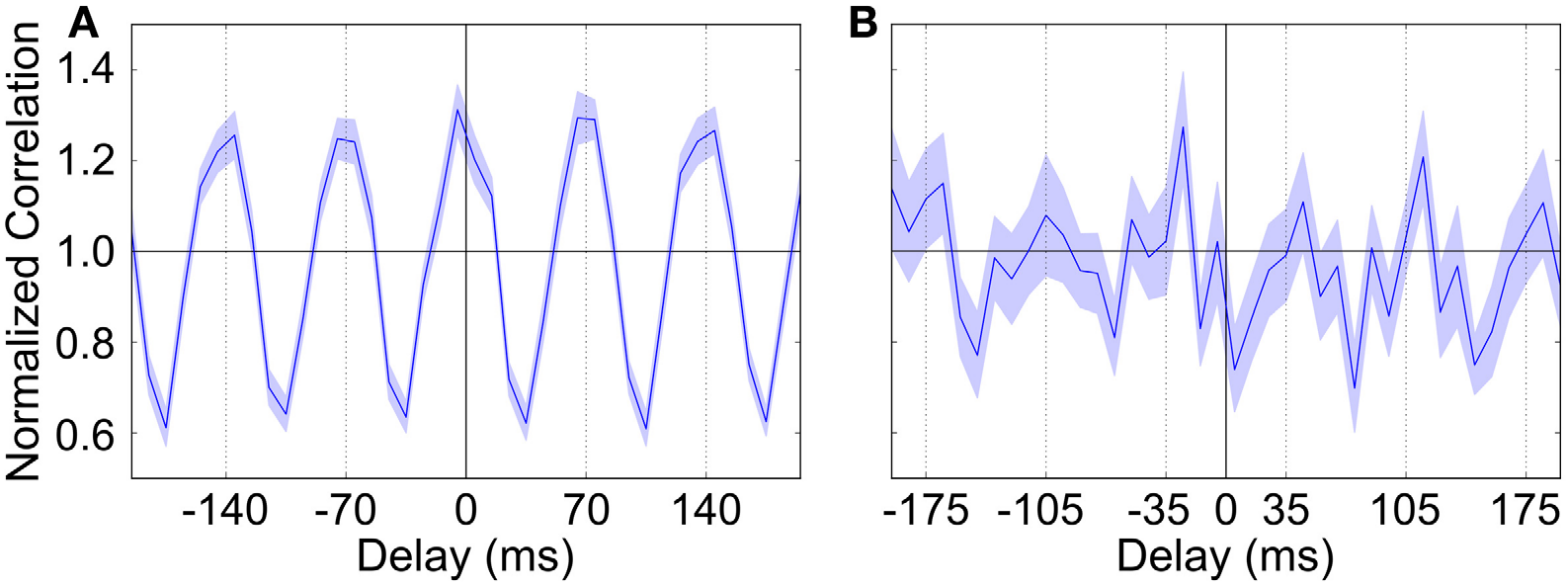
**Spike cross-correlation diagrams showing the relationship between STN and GP neuron firing when STN input is modulated at approximately 14 Hz (70 ms period). (A)** Typical average cross-correlation between all STN neurons and a single GP neuron showing synchronized in-phase firing. **(B)** STN-GP cross-correlation for a (rare) GP neuron that shows firing that is anti-phase to the β rhythm. Normalization is as in Figure 2 (Brillinger, 1976) and the shaded area shows the standard deviation across STN neurons.

We briefly investigated to see if the activity of GP neurons could become entrained to higher frequency cortical rhythms, specifically those in the β band. To do this we generated STN spike trains that were modulated at approximately 14 Hz (70 ms period: 40 ms inactive phase, 30 ms active phase). Although biological experiments on OHDA lesioned rodents find that most neurons fall under the same TI or TA category regardless of whether the cortical rhythm is SWA or β, it was difficult and not effective to use our normal method to categorize neurons because the number of spikes fired by GP neurons in each β cycle was very low. However, examining spike cross-correlations between STN and GP neurons showed that the majority of GP neurons did show oscillatory firing that was in-phase with the STN input (Figure 8A). We examined auto-correlations for individual GP neurons and found that the frequency of these neurons' oscillations varied somewhat from neuron to neuron, which suggests that their firing becomes synchronized to some intermediate frequency between the 14 Hz input and their intrinsic pacemaking frequency. We did not see any GP neurons that showed anti-phase oscillations when using our standard Parkinsonian parameters. However, when the degree of intra-GP inhibition is dramatically increased (40% coupling, *g*_*GG*_ = 3.0 nS) then a few neurons do begin to show a preference for anti-phase firing (Figure 8B), albeit at a very low rate. It is possible that with different synaptic parameters or connection topology (for example, STN input that preferentially makes contact with a particular group of GP neurons), the synchronized activity of one group could cause a second group to become synchronized in anti-phase.

## 4. Discussion

### 4.1. A new, biologically detailed, model helps us to study GP dynamics

We have presented what we believe to be a novel model of GP neurons that features much of the biological realism of previous detailed multi-compartmental models but considerably reduced complexity (both computationally and in terms of model construction). This makes our model well-suited to detailed modeling of the dynamics of networks of GP neurons and their connections with other nuclei. We have also introduced a possible computational mechanism for simulating the downregulation of HCN channels and shown that this improves how closely our results fit with biological evidence.

Our results demonstrate a mechanism whereby local inhibitory connections allow two anti-phase oscillatory subpopulations of GP neurons to emerge in response to rhythmic excitatory input from the STN. The two subpopulations appear due to a complex self-organization process and despite the homogenity of the overall population. This effect is only seen when both the STN input and inhibitory GP-GP coupling are sufficiently strong, and there is good experimental evidence that both STN input to the GP and intra-GP coupling increase in rodent models of Parkinson's disease. We therefore claim that our model shows a plausible mechanism for those experimental results which show a prominent increase in the number of TA and TI neurons that occurs in the rodent GP after dopamine lesioning (Magill et al., 2001). In our model, HCN channel downregulation makes oscillatory entrainment of the in-phase group of neurons more prominent but is not essential for the two groups to appear. This may explain the result of Chan et al. (2011) whereby artificial up-regulation of HCN channels via viral transfection restored the cells' ability to autonomously pacemake but did not give any significant improvement to Parkinsonian motor impairment. The fact that we did not see an increase in the number of neurons that were unable to autonomously pacemake following simulations of the Parkinsonian network may indicate that we didn't set the threshold for HCN downregulation low enough.

### 4.2. Relationship to previous studies on coupled oscillators

Networks of coupled oscillators are found in many areas of science and the dynamics of such networks have therefore been widely studied from a theoretical standpoint. Our model can be thought of as a network of inhibitory coupled oscillators that receive random, sparse excitatory input with a particular global frequency. Although many theoretical studies of similar systems use reduced models, they may still provide insights into the different dynamical behaviors that our model is likely to exhibit.

Most previous theoretical studies do not include common external input to the coupled oscillators, although some may consider the effects of input that is constant in time. Chow (1998) describes the analysis of a neuronal network that consists of a number of oscillators with heterogenous spiking frequencies that are all-to-all coupled by inhibitory connections. Such networks are capable of producing a range of dynamics, including almost-synchronous phase-locking, harmonic locking, and suppression. The stability of these states strongly depends on the details of the neurons' response to synaptic input. This network is similar to our model in the case where STN input is made constant in time, although inhibitory coupling in the GP network is random and relatively sparse rather than all-to-all. Since SWA oscillations are much slower than the GP neurons' intrinsic pacemaking, we can consider the GP network during the active and inactive phases separately, with constant STN input within each phase. Using the terminology of Chow (1998), during the active phase of SWA (high STN input) the TI neurons are in the suppressed state while in the inactive phase the TA neurons are suppressed. We have not observed synchrony between neurons during active or inactive phases, although it's possible that the system would converge to these states after a long period of time with constant input.

As the frequency of the cortical modulation is increased to be closer to the GP neurons' pacemaker frequencies (e.g., into the β band) it no longer makes sense to consider the scenario of constant STN input. In this scenario, theoretical results from oscillator models may be useful for suggesting conditions that support the emergence of in-phase and anti-phase groups. Golomb et al. (1992) describes a network of phase oscillators that all receive common global input that is a function of the phases of the oscillators. This is not explictly the case in our model, but nevertheless their findings regarding the stability of solutions with clustered phase distributions may be relevant. In particular they show that the fewer clusters a state has, the more stable it is (larger basin of attraction). This could explain why the GP network under β modulation organises into just two anti-phase clusters. Kilpatrick and Ermentrout (2011) study a more biologically realistic model for the emergence of gamma rhythms in a network containing a large population of excitatory neurons with a smaller subpopulation of inhibitory interneurons. Interestingly, they show that the number of clusters that emerge in their model depends on the level of spike frequency adaptation in the excitatory neurons, which arises due to a calcium current. Our model contains a calcium channel that activates during fast firing and causes some degree of spike frequency adaptation, which raises the possibility that using different conductances for this channel may result in patterns with more than two clusters. It has also been shown that networks of neurons that have heterogenous synaptic interconnectivity may display clustered dynamics if the connectivity structure satisfies certain conditions (Li et al., 2003)—although this has only been shown for excitatory synapses and so it is not clear whether the same would apply to the GP network.

### 4.3. Stimulation of the STN may reduce oscillations in the hyperdirect pathway

The hypothesis that basal ganglia activity is entrained to cortical rhythms via the hyper-direct pathway in Parkinson's disease offers some explanation of the possible mechanism(s) underlying the clinical effectiveness of STN deep-brain stimulation (DBS), in which an implanted electrode provides constant electrical stimulation of approximately 120 Hz to the STN. The precise effects of this stimulation on neuronal activity in the basal ganglia are not fully understood and are likely to be many and varied (Kringelbach et al., 2010). The computational model of the basal ganglia of Kumar et al. (2011) included the effects of DBS through either a reduction of strength of cortex-STN synapses or inhibitory input onto STN and in both cases DBS was found to reduce oscillatory firing. In this model oscillations appear because the STN and GPe act as a pacemaker circuit due to the excitatory connections between STN neurons. It is clear that if DBS were added to our model in a similar manner then oscillations in the GP would be much reduced, as they are dependent on reasonably strong input from the STN. Another possible mechanism of DBS that has good experimental support is that it antidromically activates the fibers that project from the cortex to the STN (Li et al., 2007). The effect of this antidromic activiation is a reduction of oscillatory activity in the cortical regions that project to the STN. Since our model only includes STN input to the GP, clearly a reduction of oscillatory STN activity would reduce GP oscillations as well. Wilson et al. (2011) found that in a relatively abstract model of the GP consisting of uncoupled phase oscillators synchronized to a common input, chaotic dynamics served to desynchronize the population at frequencies and intensities similar to those that are clinically effecive for DBS. The same may also be true of our model when the GP neurons are entrained to β-frequency STN input but further investigation is required. Our model could also help to test and improve the effectiveness of new forms of DBS, such as that proposed by Popovych and Tass (2012) which involves using multiple electrodes to desynchronize groups of neurons that have become entrained to particular rhythms.

### 4.4. The emergence of TI/TA groups depends on strong inhibitory coupling

One possible weakness of our model is that it relies on intra-GP inhibition being much denser than is currently supported by experimental evidence. It has been estimated that the probability of a given GP neuron synapsing onto any other, randomly-chosen, GP neuron is less than 1% (Sadek et al., 2007) but in most of our simulations this value is 20%. Our preliminary experiments with larger networks have suggested that the level of intra-GP coupling can be reduced while preserving the division into TI and TA groups by increasing the size of the network. Further work will involve investigating even larger (more computationally expensive) networks to see how much further the size of the network can be increased while maintaining the same division.

If further increases in network size cannot generate realistic activity with physiological levels of GP-GP coupling, there are several other possible reasons why the connectivity may be greater than has so far been measured experimentally. It has been suggested that the basal ganglia are organized into a series of partially overlapping “channels” (Alexander and Crutcher, 1990), where neurons preferentially synapse onto other neurons in the same channel. We have previously shown modeling evidence that increased coupling between channels may allow the STN-GP circuit to generate oscillations (Merrison-Hort et al., 2013), but in the present study we suggest that our small population of GP neurons could represent part of a single channel. Under this assumption, the proportion of coupled neurons might be much higher than would be seen from picking pairs of neurons from across the whole GP at random. It is also possible that the projection from GP to STN, which is not included in our model, may contribute to the effect of lateral inhibition since the tri-synaptic GP-STN-GP pathway is a route by which GP neurons inhibit other GP neurons, and there is experimental evidence to suggest the strength of this pathway may be increased under Parkinsonian conditions (Johnson and Napier, 1997). However, it is hard to say whether or not this explanation is plausible without more detailed information about the topology of the STN-GP and GP-STN projections.

Similarly, the increase in GP-GP synaptic conductance that occurs under Parkinsonian conditions in our model may be larger than in reality. Although we have used data from paired-pulse experiments to choose the conductance of GP-GP synapses in the healthy case, it is not clear how much this increases by following loss of dopaminergic input. Miguelez et al. (2012) used an optogentic technique to stimulate a number of GP neurons whilst recording IPSCs and found an increase of approximately 67% after dopamine lesioning. This is considerably smaller than the increase we use under Parkinsonian conditions, where the GP-GP conductance is doubled. However, the increase seen by Miguelez et al. (2012) may be lower than the in conductance at a single GP-GP synapse, since their method simultaneously activates many pre-synaptic GP neurons and the summation of the resulting IPSCs may not be linear.

### 4.5. Future work: striatal input, reciprocal connections and GP heterogenity may improve our results

The aim of this study was primarily to investigate whether the hyperdirect pathway alone could account for one characteristic of the Parkinsonian GP and we have therefore only included STN-GP and GP-GP synaptic connectivity. However, the main source of synaptic input to the GP is the striatum, and it is clear that adding simulated inhibitory striatal synaptic input could improve our results. Galati et al. (2009) demonstrated that the delivery of a GABA_A_ antagonist into the GP also causes cortical entrainment of the neurons there and that this effect is dependent on a functioning STN. They suggest that this demonstrates that inhibitory striatal input is also involved in oscillatory entrainment. This result is more difficult to explain in our model, since it is unlikely that decreasing the level of GP-GP inhibition would cause oscillations to appear in the (otherwise) healthy case. However, if the effect of GABA antagonism is to remove *tonic* background inhibition (probably from the striatum) then we could include this in our model as a depolarizing current injection to all GP neurons. This would move their membrane potentials closer to the spike threshold and make them more sensitive to the (weak) rhythmic STN input that is present in the healthy case. Furthermore, Tseng et al. (2001) showed that the activity of striatal projection neurons is modulated by cortical SWA and increases after OHDA lesion. Including the effects of this in our model would probably increase the number of TI neurons and may allow us to reduce the amount of intra-GP inhibition to a more realistic level.

Another possible pathway that could be added to our model is the projection from the GP back to the STN. Computational models of networks that include this connection have shown that the STN and GP can work together to act as a pacemaker circuit (Gillies et al., 2002; Terman et al., 2002; Holgado et al., 2010). Terman et al. (2002) describes the results of simulating a spiking model of the interconnected STN-GPe network in which the tonic activity of both populations can become bursty with a regular bursting rhythm. In fact, the neurons in this model can self-organise into different sized clusters, which allows for the possibility of two anti-phase groups under some conditions. We expect that our model could support similar regimes if the GP-STN connection was added, provided that we also introduced a more realistic STN neuron model.

Although our model has demonstrated that discrete groups of neurons can emerge from a population of GP neurons with homogeneous (unimodally distributed) membrane properties, there is now some evidence that the neurons in the TA and TI groups are distinct in some ways, including the nature of local inhibitory connectivity, the basal ganglia nuclei that they project to, and in their chemistry (Mallet et al., 2012). Similarly, several studies (Nambu and Llinás, 1997; Cooper and Stanford, 2000; Bugaysen et al., 2010) have attempted to categorize GP neurons based on their electrophysiological properties, and their results seem to suggest that several distinct groups may exist (although the boundaries remain fuzzy). It would be very interesting to incorporate these results into our model, perhaps by making the parameter noise for the NaP or HCN channels bi- or tri-modal and by giving one group of neurons a higher degree of local connectivity than another. We expect that this would promote the emergence of the TI, TA and NM groups and would probably reduce the degree of GP-GP connectivity that is required in order to obtain results that are similar to experiments.

## Conflict of interest statement

The authors declare that the research was conducted in the absence of any commercial or financial relationships that could be construed as a potential conflict of interest.
